# Bilateral strength asymmetry in knee extension: a reliability and consistency analysis for scientific and practical applications

**DOI:** 10.3389/fspor.2025.1635685

**Published:** 2025-09-16

**Authors:** Manfred Zöger, Alfred Nimmerichter, Arnold Baca, Klaus Wirth

**Affiliations:** ^1^ Training and Sports Sciences, University of Applied Sciences Wiener Neustadt, Wiener Neustadt, Austria; ^2^ Centre for Sport Science and University Sports, University of Vienna, Vienna, Austria; ^3^ Doctoral School of Pharmaceutical, Nutritional and Sport Sciences, University of Vienna, Vienna, Austria

**Keywords:** bilateral strength asymmetry, dynamometry, knee extension, muscle imbalance, reliability

## Abstract

**Background:**

Muscular strength imbalances are associated with musculoskeletal injuries and performance deficits. This study aimed to assess the reliability and consistency of bilateral strength asymmetry (BSA) in quadriceps muscles as determined by dynamometry.

**Methods:**

Twenty-nine physically active participants underwent a familiarization and two test sessions, assessing isometric (100°, 140° knee angles) and isokinetic (30° s^−1^, 400° s^−1^) knee extension. BSA was calculated from Peak torque (PT), and reliability was evaluated using paired *t*-test, intraclass correlation coefficients (ICC), standard error of measurement (SEM), minimum detectable change (MDC), and Kappa coefficients for limb dominance consistency. Bland-Altman plots assessed agreement between sessions.

**Results:**

No significant differences in mean PT or BSA were found between sessions. However, considerable individual variability was observed, with 21%–38% of participants showing between session limb dominance reversals. ICC values ranged from 0.33 to 0.70 (poor to moderate reliability), SEM from 5.0% to 8.3%, and MDC from 13.9% to 22.9%. Kappa coefficients indicated less than chance to moderate agreement for limb dominance between sessions (0.19–0.59) and within sessions (−0.05–0.50). Bland-Altman analyses revealed small bias but wide limits of agreement.

**Conclusions:**

BSA measurements demonstrated low reliability and inconsistency, with significant individual variability and frequent limb dominance reversals. These findings raise concerns about the use of fixed asymmetry thresholds for injury risk assessment. Task-specificity and individual motor control variations may contribute to these inconsistencies. Future studies should consider habituation and standardized training protocols before evaluating BSA reliability. The results emphasize the need for cautious interpretation of BSA data and highlight limitations in its direct clinical application.

## 1 Introduction

Muscular imbalances have been consistently identified as a significant risk factor contributing to a wide range of musculoskeletal injuries and re-injuries (1–7). Several prospective and retrospective studies have shown a correlation between imbalances with hamstring and knee injuries, as well as unexplained falls in the elderly (2, 6, 8–11). Also in performance sports, research into limb strength imbalances is gaining attention, as asymmetries can negatively impact optimal performance (12, 13).

Given the potential for imbalances to impede sporting performance, increase the risk of injury and compromise rehabilitation outcomes, accurate assessment is crucial for developing targeted balancing strategies and optimizing recovery. Furthermore, this is important in the context of return-to-sport or competitive participation after an injury, where decisions frequently take into account the extent of remaining strength imbalances (14–17).

Most available sources suggest strength asymmetry thresholds ranging from 10% to 15%, depending on study design and methodology (2, 18–21). However, some studies report thresholds as high as 20% (22) for an increased risk of injury, while other suggest values as low as 2%–5% for a criterion to discharge from treatment after knee injury (23). Nevertheless, no consensus exists regarding a critical boundary for strength imbalances in the lower extremities as these commonly used thresholds are not robustly supported by literature (24, 25).

A possible explanation for this discrepancy could be differences in testing methodologies (26). Commonly used methods for assessing bilateral asymmetries include isokinetic tests [the most common (27)], isometric tests, squats, and various jump tests (28). However, the generalizability of findings regarding strength imbalances across diverse testing conditions is limited, as asymmetries seem to be task-specific (7, 27, 29).

For example, Impellizzeri et al. (27) found only moderate correlations (*r* = 0.48) between strength asymmetries in isokinetic leg extension at 60° s^−1^ and 240° s^−1^ and a vertical jump force test. Another similar study by Menzel and colleagues (29) also analyzed lower limb asymmetries for vertical jump performance parameters and isokinetic knee extension. They found only low to moderate correlations in the range of *r* = 0.19–0.46 for the comparison of isokinetic peak torque and variables of the counter movement jump. A study conducted by Newton et al. (7) indicated non-significant relationships for the comparison of squat force and isokinetic knee extension at 60° s^−1^ and 240° s^−1^. The observed correlations ranged from *r* = 0.02–0.49. It should be noted here that isokinetic knee extension represents an open kinetic chain movement, whereas vertical jump and squat tests are performed in a closed kinetic chain. Differences in the type of kinetic chain my contribute to the low to moderate correlations observed in these studies.

While the importance of addressing muscular strength imbalances is well-established, consensus on optimal assessment methods and critical thresholds remains lacking. Despite numerous studies comparing asymmetries across various tasks (e.g., squats, jumps, isokinetic tests), few have examined the reliability of quadriceps strength asymmetry under both isometric and isokinetic conditions.

To the best of the authors knowledge, only one study to date (30) has investigated the reliability of strength asymmetry in healthy subjects using isokinetic leg extension measurements. However, no study has yet compared isokinetic and isometric strength asymmetries using a dynamometer, despite its status as the gold standard for muscle force testing (31). Therefore, this study aims to address this gap by comparing quadriceps strength asymmetries at multiple knee angles and velocities in a test-retest design, using a dynamometer to assess both slow (30° s^−1^) and fast (400° s^−1^) concentric knee extension as well as isometric knee extension at 100° and 140° of knee angle. The target of this approach was to examine whether muscular strength asymmetries are reliable and consistently favor the same limb during unilateral single-joint dynamometric tests.

## 2 Materials and methods

### 2.1 Subjects

Twenty-nine physically active subjects (mean (± SD): stature 177.7 (8.7) cm; body mass 74.8 (10.0) kg; age 26.4 (6.1) years) without a documented history of orthopedic lower extremity conditions agreed to participate in this research. All individuals were physically active at a recreational level but had no prior experience with isokinetic exercises. 24 of the subjects were right-limb preferred, defined via the limb they would use to kick a ball (32). To ensure consistent testing conditions, participants were instructed to abstain from strenuous physical activity for 48 h, caffeine intake for 12 h, and food consumption for 3 h prior to each test. Before their initial visit to the laboratory, subjects were informed about the potential benefits and risks associated with participating in the study. All participants provided written informed consent and were advised that they had the option to withdraw from the study at any time. The study was approved by the local research ethics board at the University of Applied Sciences Wiener Neustadt on the 5th of April 2021 (approval nr RB20210405013) and adhered to the ethical principles outlined in the Declaration of Helsinki (33).

### 2.2 Instruments

The IsoMed 2000-dynamometer (D. & R. Ferstl Gmbh, Hemau, Germany) equipped with the manufacturer's unilateral knee attachment was used for conducting the experiments within this study. The device was calibrated prior to each testing session according to the manufacturer's instructions. Data acquisition was performed at a sampling rate of 200 Hz using the manufacturer's IsoMed analyze SP3-i51 software.

### 2.3 Procedures

As recommended by several authors (34–36), all subjects started with a familiarization session prior to the experimental task, to become acquainted with the device, isokinetic exercise itself, and the test protocol. This familiarization session was conducted identically to the subsequent experimental sessions, ensuring that participants were fully acquainted with the characteristics of isokinetic exercise and test procedures, before commencing the main sessions. The familiarization session was typically conducted 72 h before the first main session, with a minimum of 48 h between sessions, to allow for sufficient recovery in between. All sessions were conducted by the same experimenter to avoid inter-tester variability.

Subjects started with a standardized general warm-up, consisting of 10-min cycling on a stationary ergometer at a submaximal intensity of 1.5 W per kg bodyweight and a cadence of around 70 rpm. Following this general warm-up, participants were seated in the adjustable dynamometer chair with the backrest positioned at 85° (0° = fully extended). The popliteal fossa of the tested leg was aligned with the frontal edge of the seat. The knee's rotational axis was aligned with the dynamometer's mechanical axis using a laser pointer, with the lateral femoral epicondyle serving as a bony reference point. Adjustable straps and padding across the shoulders, hip, and femur were utilized to reduce extraneous body movements, enhance stabilization and to isolate knee joint movement. Participants were also instructed to hold onto the side handles of the device.

The dynamometer lever arm and the corresponding distal shin pad were secured approximately 2.5 cm above the lateral malleolus with a strap, while the knee was in a position of 90° flexion. The knee joint range of motion was restricted to 90°–170° (180° = fully extended). Following the correct positioning of the participants, individual settings for each subject were recorded by the integrated software, to ensure identical placement for both the familiarization and experimental sessions.

The test protocol included four unilateral knee extension conditions performed with both legs, with a random selection of the starting leg. However, right- and left-leg starts were equally distributed across the participant group. Regarding the four conditions, two of them were executed in isometric mode and two in isokinetic mode. For the isometric measurements, the knee joint was fixed at angles of 100° (Iso100) and 140° (Iso140). Isokinetic measurements were executed at angular velocities of 30° s^−1^ and 400° s^−1^, with the slower velocity preceding the faster velocity, as suggested by a previous study (37). All isokinetic measurements were executed as single-direction, discrete movements and the initial starting position was achieved passively. The order for the test protocol remained the same for the familiarization and the experimental session and was Iso100, Iso140, 30° s^−1^, 400° s^−1^.

Before each condition, participants completed a submaximal specific warm-up exercise on the device to become accustomed to the demands of each test. This specific warm-up consisted of 10 repetitions at an intensity corresponding to approximately 50% of maximum voluntary contraction followed by 3 repetitions at an intensity corresponding to approximately 80% of maximum voluntary contraction. The specific warm- up was followed by a 3-min break where the exact procedures for the following condition were explained by the examiner via standardized instructions.

A minimum of three repetitions were completed for each testing condition. However, additional repetitions were conducted until peak torque (PT) no longer increased. All subjects reached PT within a maximum of five repetitions. After each repetition, subjects received 3-min of passive rest for sufficient recovery before starting the next repetition. To maximize effort, participants were provided with visual feedback on a screen and additional strong verbal encouragement from the examiner. Following each test condition, the dynamometer's position was adjusted for the opposite leg or the subsequent condition.

### 2.4 Interlimb asymmetry calculation

Relative lower limb strength asymmetry has previously been calculated in several ways (28). We used the formula for the calculation of bilateral strength asymmetry (BSA) (7, 38):

$$\mathrm{BSA}\;(\text{\%})=\frac{\left(\mathrm{stronger}\;\mathrm{limb}\text{-}\mathrm{weaker}\;\mathrm{limb}\right)}{\mathrm{stronger}\;\mathrm{limb}}*100$$

One potential shortcoming of this formula is that it always results in positive values. This is a problem when comparing asymmetries for the same subjects while performing different test modalities, as there is the possibility that the stronger limb will become the weaker one in a subsequent condition. Using absolute BSA values while disregarding their direction would not permit a valid comparison of different diagnostic methods. This is because different modalities might indicate opposite dominance with the same magnitude. Consequently, such an approach could lead to the erroneous conclusion that both methods yield identical results, although they actually produce opposite values. To overcome this disadvantage, we decided to still use the BSA formula as mentioned above, but modify the result according a previous study (27). Impellizzeri and colleagues arbitrarily assigned a negative sign (–) when the left leg was the stronger one. That said, positive BSA values imply a stronger right leg while negative BSA values display a stronger left leg.

### 2.5 Statistical analysis

All data are presented as mean ± SD. For each condition, the repetition with the highest PT for the knee extensors was selected for further analysis (39, 40). The corresponding PT-values were used for calculating BSA (%) according to the formula mentioned above. The assumption of normality was verified using Shapiro–Wilk test.

Noting that asymmetries can favor either the right or left limb, a Kappa coefficient (41) was calculated to determine the consistency of limb dominance across test conditions and sessions and interpreted according to suggestions from Viera and Garrett (42). These recommendations classify kappa values as follows: <0 = less than chance agreement, 0.01–0.20 = slight agreement, 0.21–0.40 = fair agreement, 0.41–0.60 = moderate agreement, 0.61–0.80 = substantial agreement and 0.81–0.99 = almost perfect agreement.

Paired sample *t*-tests were conducted to determine systematic bias and to quantify whether asymmetry scores were significantly different between sessions. The magnitude of the difference between sessions was determined using Cohen's d effect sizes, calculated using the formula (43, 44):

$$d=\frac{M_{\mathrm{diff}}}{S_{\mathrm{pooled}}}$$

where M_diff_ is the mean difference between repeated measures and S_pooled_ is the pooled SD of the measurements at each time point. These effect sizes were interpreted according to Cohen's guidelines (45), with |d| ≥ 0.20 indicating a small effect, |d| ≥ 0.50 a medium effect, and |d| ≥ 0.80 a large effect.

A two-way random-effects intraclass correlation coefficient (ICC) was calculated to assess relative reliability. These results were interpreted following the recommendations of Koo and Li (46). According to these guidelines, an ICC above 0.9 indicates excellent reliability, values between 0.75 and 0.9 are considered good, values between 0.5 and 0.75 are deemed moderate and an ICC below 0.5 is indicative of poor reliability. Beside relative reliability, absolute reliability was also evaluated by calculating the standard error of measurement (SEM) using the formula (47, 48):

$$\mathrm{SEM}=\mathrm{SD}\;*\;\sqrt{\;1\text{-}\mathrm{ICC}}$$

To determine whether a difference between two measurements in a subject was real, the minimum detectable change (MDC) was calculated. MDC was computed based on a 95% Confidence interval (CI) about the SEM, following the formula described by Weir (48):

$$\mathrm{MDC}=\mathrm{SEM}\;*\;1.96\;*\;\sqrt{\;2}$$

To assess agreement between sessions, Bland-Altman statistics with ±95% limits of agreement (LoA) were computed, and corresponding plots were generated to visually represent individual differences.

All statistical analyses were conducted using IBM SPSS Statistics, version 29.0.1 (IBM Corp., Armonk. NY, USA). Figures were created with GraphPad Prism, version 10.4.1 for windows (GraphPad Software, San Diego, CA, USA). The level of significance was set at *p* < 0.05.

## 3 Results

Mean values (± SD) of PT and BSA for session 1 and session 2, including *p*-values and Cohens *d* Effect sizes are presented in Table 1. No significant differences were observed between sessions for either PT or BSA. Mean BSA was in the range of −0.4–3.1 for session 1 and in the range of −0.7–2.7 for session 2. Effect sizes for the variations in BSA between sessions ranged from 0.02 to 0.29, indicating small to negligible effects.

**Table 1:** Mean (± SD) for peak torque and bilateral strength asymmetry in session 1 and session 2 as well as *p*-values and Cohen's *d* effect size for comparison of sessions.

	Session 1	Session 1	Session 1	Session 2	Session 2	Session 2	PT main effect	BSA main effect	BSA
	PT (Nm)	PT_rel_ (Nm·kg^−1^)	BSA (%)	PT (Nm)	PT_rel_ (Nm·kg^−1^)	BSA (%)	*p*-value	*p*-value	ES
Iso100 R	249.9 (±50.0)	3.35 (±0.58)	2.5 (±9.3)	256.0 (±50.5)	3.42 (±0.47)	2.7 (±9.2)	0.21	0.09	0.02
Iso100 L	244.9 (±60.2)	3.26 (±0.60)		250.7 (±59.4)	3.33 (±0.55)		0.20		
Iso140 R	244.2 (±50.2)	3.27 (±0.53)	2.1 (±7.8)	246.0 (±56.1)	3.28 (±0.55)	0.2 (±7.5)	0.58	0.31	0.24
Iso140 L	240.0 (±55.5)	3.20 (±0.51)		246.9 (±62.3)	3.29 (±0.61		0.08		
30° s^−1^ R	229.6 (±47.9)	3.07 (±0.47)	3.1 (±9.6)	231.1 (±50.5)	3.08 (±0.50)	0.4 (±9.0)	0.68	0.18	0.29
30° s^−1^ L	222.5 (±51.2)	2.97 (±0.47)		230.4 (±53.4)	3.07 (±0.48)		0.07		
400° s^−1^ R	100.6 (±29.6)	1.33 (±0.30)	−0.4 (±11.2)	102.3 (±30.4)	1.35 (±0.28)	−0.7 (±10.6)	0.21	0.92	0.02
400° s^−1^ L	100.7 (±28.8)	1.33 (±0.27)		102.8 (±29.3)	1.36 (±0.27)		0.35		

BSA, bilateral strength asymmetry; PT, peak torque; Nm, Newton meter; PT_rel_, peak torque normalized per kg body mass; ES, effect size; Iso100, isometric 100° knee angle; Iso140, isometric 140° knee angle; R, right leg; L, left leg.

**Figure 1:**
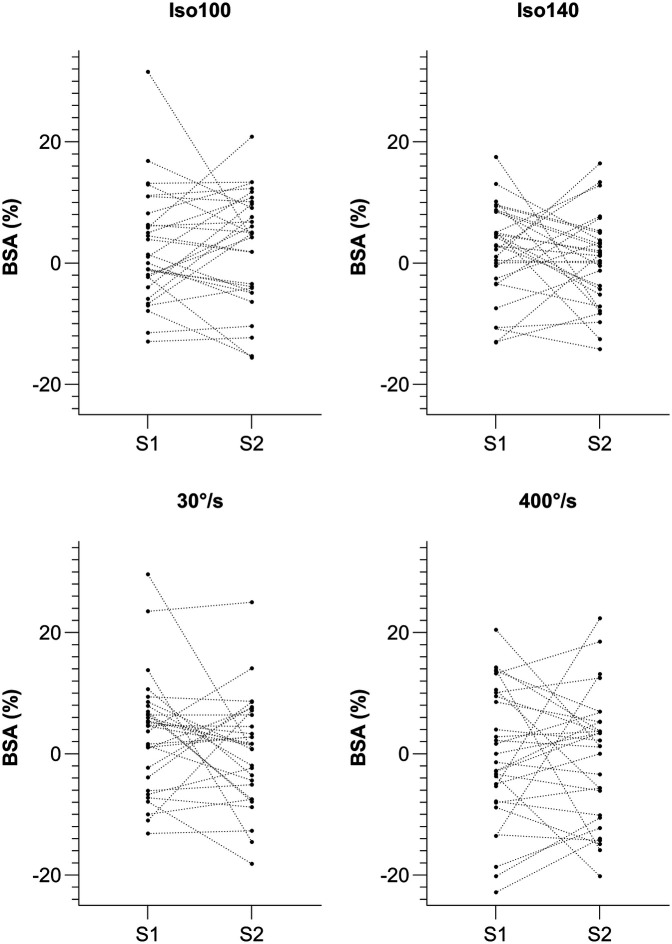
Individual alterations in BSA between test session 1 and 2 for Iso100, Iso140, 30° s^−1^ and 400° s^−1^. Isometric extension 100° knee angle, Isometric extension 140° knee angle, Isokinetic extension 30° s^−1^ and Isokinetic extension 400° s^−1^.

**Figure 2:**
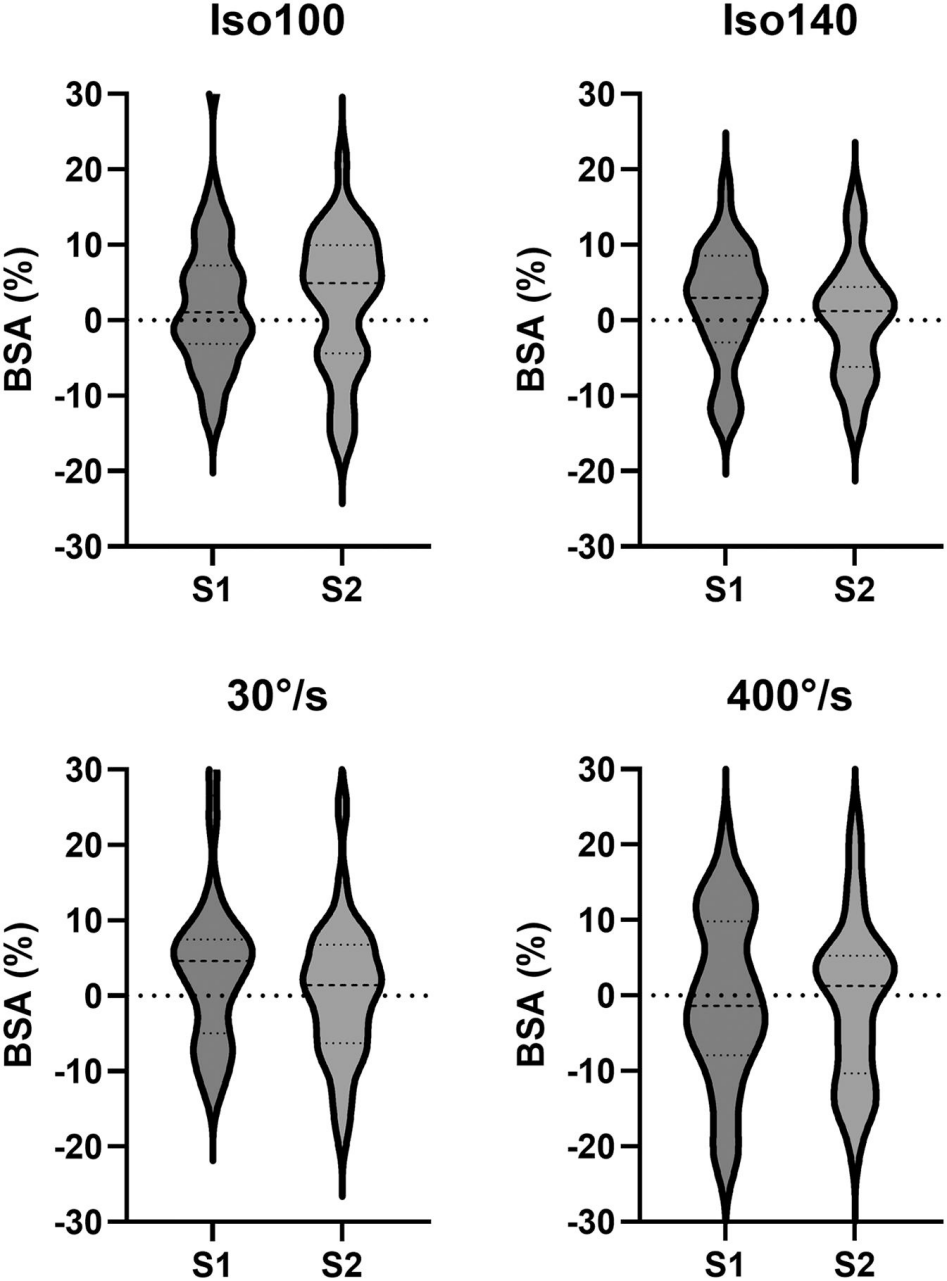
Violin-plots – distribution of BSA for session 1 and 2 for Iso100, Iso140, 30° s^−1^ and 400° s^−1^. Isometric extension at 100° knee angle; Isometric extension at 140° knee angle, Isokinetic knee extension at 30° s^−1^ and Isokinetic knee extension at 400° s^−1^.

Nine participants (36%) did not show a reversal of limb dominance between S1-S2 within the same condition, with five of them (20%) showing a reversal of limb dominance between at least one of the conditions. This results in four subjects (16%) consistently favoring the same limb across all four conditions and in both sessions. The alterations in BSA across test sessions, along with levels of agreement in limb dominance are presented in Table 2. Results for kappa coefficient comparison of sessions showed slight to moderate levels of agreement (range = 0.19–0.59). Individual alterations in BSA between test sessions are presented in Figure 1. The distribution of BSA for several conditions and in both sessions are presented in Figure 2, using Violin-plots.

**Table 2:** Bilateral strength asymmetry changes and kappa coefficient including descriptive level of agreement showing how consistently asymmetry favors the same leg between session 1 and session 2.

	Subjects (*n*, %) with a BSA change >10% between S1–S2	Subjects (*n*, %) with a reversal of limb dominance between S1–S2	Subjects (*n*, %) with a reversal of limb dominance between S1–S2 including a BSA change >10%	Kappa coefficient	Descriptor
Iso100	7 (24.1%)	6 (20.7%)	3 (10.3%)	0.58	Moderate
Iso140	7 (24.1%)	11 (37.9%)	5 (17.2%)	0.19	Slight
30° s^−1^	11 (37.9%)	9 (31.0%)	7 (24.1%)	0.34	Fair
400° s^−1^	7 (24.1%)	6 (20.7%)	4 (13.8%)	0.59	Moderate

BSA, bilateral strength asymmetry; S, session; Iso100, isometric 100° knee angle; Iso140, isometric 140° knee angle.

The results for the kappa coefficient within sessions comparison of limb dominance are presented in Table 3. They showed slight to fair levels of agreement within session 1 (0.05–0.36), with an exception between Iso100 and Iso140 (−0.05) that showed less than chance levels of agreement. For session 2, all comparisons that included the Iso100 condition increased to the levels of fair to moderate (0.27–0.50), while the others decreased or remained at the same level of slight agreement (0.09–0.15).

**Table 3:** Kappa coefficient including descriptive level of agreement showing how consistently asymmetry favors the same leg within sessions 1 and 2, respectively.

		Kappa coefficient	Descriptor
Session 1			
Iso100	Iso140	−0.05	Less than chance
Iso100	30° s^−1^	0.23	Fair
Iso100	400° s^−1^	0.24	Fair
Iso140	30° s^−1^	0.36	Fair
Iso140	400° s^−1^	0.18	Slight
30° s^−1^	400° s^−1^	0.05	Slight
Session 2			
Iso100	Iso140	0.27	Fair
Iso100	30° s^−1^	0.42	Moderate
Iso100	400° s^−1^	0.50	Moderate
Iso140	30° s^−1^	0.15	Slight
Iso140	400° s^−1^	0.09	Slight
30° s^−1^	400° s^−1^	0.09	Slight

Iso100, isometric 100° knee angle; Iso140, isometric 140° knee angle.

The results for ICC in the range of 0.33–0.70 indicate poor to moderate relative reliability (95% CI −0.42–0.86). Absolute reliability expressed as SEM revealed values of 5.0%–8.3%. The MDC that was calculated to determine if a difference between two measurements on a subject can be considered real, was in the range of 13.9%–22.9% (Table 4).

**Table 4:** Relative and absolute reliability statistics for bilateral strength asymmetry including intraclass correlation coefficient and 95% confidence interval, standard error of measurement, minimum detectable change and bland-altman statistics including bias (±SD) and 95% limits of agreement for comparison of session 1 and session 2.

	ICC	ICC 95% CI	SEM	MDC	Bias (±SD)	95% LoA
Iso100	0.70	0.36–0.86	5.0	13.9	−0.2 (±9.0)	−17.7–17.4
Iso140	0.33	−0.42–0.68	6.3	17.4	1.9 (±9.7)	−17.1–20.9
30° s^−1^	0.51	−0.02–0.77	6.5	18.1	2.7 (±10.6)	−18.1–23.5
400° s^−1^	0.42	0.11–0.81	8.3	22.9	0.2 (±11.9)	−23.1–23.5

ICC, intraclass correlation coefficient; CI, confidence interval; SEM, standard error of measurement; MDC, minimum detectable change; SD, standard deviation; LoA, limits of agreement; Iso100, isometric 100° knee angle; Iso140, isometric 140° knee angle.

**Figure 3:**
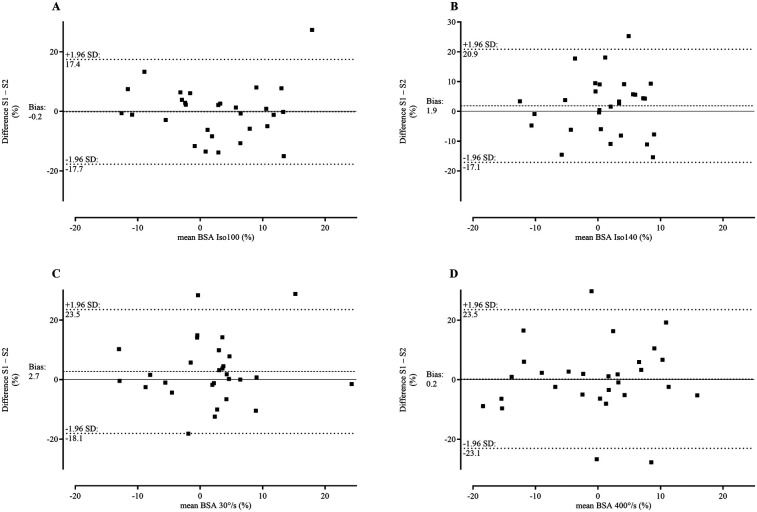
Bland-Altman plots – differences between BSA in session 1 and 2 plotted against the means of session 1and 2. Bland-Altman plots for: **(A)** Isometric extension at 100° knee angle; **(B)** Isometric extension at 140° knee angle; **(C)** Isokinetic knee extension at 30° s^−1^ and **(D)** Isokinetic knee extension at 400° s^−1^.

Graphical illustrations for subject-specific session differences compared with session averages are represented using Bland-Altman plots (Figure 3). The average difference between sessions, that is represented by the bias, is in the range of −0.2%–2.7% (95% LoA from −23.1% to 23.5%).

## 4 Discussion

This study aimed to investigate whether muscular strength asymmetries are consistent and reliably favor the same limb in unilateral single-joint dynamometric assessments. The results of this investigation indicate that BSA is inconsistent, has low reliability, and in some cases does not favor the same limb. This suggests that BSA may vary not just across different but also within the same testing conditions and should be interpreted with caution.

Establishing the reliability of muscular strength asymmetries derived via dynamometric measurement procedures is a fundamental prerequisite for their valid application in research and clinical practice. However, despite the prevalent use of asymmetry indices, the existing literature reveals a notable paucity of studies investigating their reliability.

Regarding the between session comparison in our study, we found no significant difference in PT, indicating that the subjects did not show strength improvement from session 1 to session 2. That is in line with the results of Impellizzeri, Bizzini (30), who also found no significant differences after an initial familiarization session. There was no significant difference in BSA between sessions, which again is in accordance with the results in the referenced study (30). However, the mean and SD values of BSA in our study revealed that the SD was consistently and substantially greater than the mean across all conditions and both sessions, suggesting a high degree of variability in BSA (Table 1). In terms of BSA computation, readers should keep in mind that we used the calculation method presented by Impellizzeri, Rampinini (27), where a negative sign (–) indicates a stronger left leg and a positive sign indicates a stronger right leg. Taking this into account, it is possible that a subject with a right-dominance in session 1 could have a left-dominance in session 2, while another subject represents exactly the opposite trend. In the end, a between session group change of BSA would then be nullified. When looking at individual results (Figure 1), a reversal of limb dominance was indeed observed in 21%–38% of our subjects, in all four conditions (Table 2).

Regarding relative reliability, ICC values ranging from 0.33 to 0.70 have been observed in our study, indicating only poor to moderate reliability for BSA. The generally broad 95% CI, spanning from −0.42 to 0.86, further underscores the problematic reliability, with some conditions even showing negative CI values, highlighting the uncertainty of BSA reliability. Comparatively, Impellizzeri, Bizzini (30) examined the reliability of strength imbalance ratios derived from isokinetic knee extension measurements at three different velocities. Their results showed ICC values of 0.78 for 60° s^−1^, 0.63 at 120° s^−1^, and 0.43 at 180° s^−1^, suggesting a decline in reliability as velocity increased. In contrast, Hsu, Tang (49) investigated muscle strength deficit measures and found ICC values ranging from poor to good reliability. Specifically, they reported an ICC of 0.42 for isokinetic knee extension at a slower velocity (30° s^−1^) and 0.81 at a faster velocity (90° s^−1^), indicating improved reliability at higher speeds. However, their study was conducted with a sample of nine stroke patients, which limits the generalizability of these findings.

With regards to absolute reliability, SEM in our study reached 5%–8.3%. That is similar to, though still higher than the SEM results found by Impellizzeri, Bizzini (30), that were in the range of 3.2–6.5%. In addition, a 95% interval about the SEM was used to calculate MDC to determine the smallest difference that can be considered significant with a given level of confidence, that is, the difference that is attributable to a true change and not to measurement error. The observed MDC values ranged from 13.9% to 22.9%, highlighting the need for caution when using fixed thresholds of asymmetries to assess injury risk or termination of treatment following injuries. Although prevalent in past research (2, 18–21, 23), this approach of using asymmetry thresholds has been scrutinized in a recent study (50).

The results for the Kappa coefficients and the corresponding level of agreement between identical conditions across session 1 and 2 are presented in Table 2, while within-session coefficients for various conditions are shown in Table 3. The Kappa coefficient quantifies the proportion of agreement between two methods while accounting for chance agreement (41). Given the aim of assessing the consistency of asymmetries occurring in the same limb, the observed Kappa values indicate slight to moderate levels of agreement, ranging from 0.19 to 0.59 for PT asymmetries across identical conditions in sessions 1 and 2 (Table 2). For example, when asymmetry favored the right limb during Iso140 in session 1, it was unlikely that the right limb was also favored in session 2 (Kappa = 0.19). It is important to emphasize that Kappa values incorporate a correction for agreement due to random occurrence.

For the comparison of limb agreement between several conditions within the same session, Kappa coefficients are even worse (Table 3), ranging from −0.05 to 0.50. Keeping in mind that a negative Kappa value indicates that agreement between these methods is worse than what would be expected by chance. In other words, within session 1, Iso100 and Iso140 disagreed more often than would be expected if classifications were made randomly.

The systematic differences, represented by the bias in the Bland-Altman analysis (Table 4), indicate a small discrepancy between methods (−0.2–2.7). However, the broad SD for the bias and the wide 95% LoA again suggest high variability in individual measurements (Figure 3).

Although BSA group mean values appear to be free from systematic bias, the data in general demonstrate substantial individual variability (Figures 1–3). This observation is consistent with the findings of Bishop, Read (51), and raises fundamental questions about the reliability of BSA measurements. It seems that the high variability of values complicates the interpretation of BSA data. However, the primary issue appears not to be the magnitude of BSA values themselves but rather the substantial variability, including instances of side-to-side reversals, which makes it difficult to determine critical thresholds or even calls their validity into question.

The lack of reliability in strength asymmetry ratios, previously reported by Impellizzeri (30), has been confirmed by our current data. Bishop and colleagues (52) concluded that “asymmetries are task-specific, highly individual in nature, and rarely favor the same limb when comparing across tests”. This observation is consistent with our findings. Furthermore, our results extend these insights by demonstrating that asymmetries, even within the same task (knee extension) and using the same metric (PT), lack sufficient test-retest reliability when comparing within identical conditions.

Despite these findings, readers should consider a few limitations. First, this study focused on uninjured physically active subjects, meaning that the results may not be fully applicable to other populations like untrained persons, elite athletes or individuals dealing with injuries. Second, our study used the repetition with the highest PT value obtained during several repetitions for further analysis. Other studies in the past have used a method where the average PT from several repetitions was used for further analysis (7, 27). Using this method in our study might have resulted in slightly different findings. However, there is no clear consensus in the literature on what method to use best, as our approach that used highest PT was also already used by several other authors in the past (29, 30, 39, 40, 49).

One potential factor to consider is habituation to the testing conditions. It is possible that the variability of BSA would decrease after an extended adaptation period of several weeks or months. This would, however, imply that reliable BSA measurements can only be conducted under well-habituated conditions. Nevertheless, the possibility of firmly established, immutable motor programs influencing this variability remains. At present, it is inconceivable to determine the extent of such an effect.

### 4.1 Implications

The present study highlights the inconsistency and limited reliability of BSA measurements in unilateral dynamometric assessments. Despite the widespread use of imbalance indices, our results emphasize substantial variability in BSA values, notably including frequent reversals in limb dominance between sessions. Although the group mean values showed no systematic bias, the substantial range of individual fluctuations suggests that BSA should be interpreted with caution. Both relative and absolute reliability metrics support these concerns, as ICC demonstrated only poor to moderate reliability, while SEM and MDC indicate substantial variability. These results challenge the validity of employing fixed asymmetry thresholds for injury risk assessment or clinical decision making. Future research should therefore implement a standardized training intervention across multiple sessions before conducting further reliability and validity studies. This approach could contribute to a more robust understanding of BSA, ultimately enhancing its clinical applications and the assessments of injury risks.

## Data Availability

The raw data supporting the conclusions of this article will be made available by the authors, without undue reservation.
